# Very high HIV prevalence and incidence among men who have sex with men and transgender women in Indonesia: a retrospective observational cohort study in Bali and Jakarta, 2017–2020

**DOI:** 10.1002/jia2.26386

**Published:** 2024-10-24

**Authors:** Brigitta Dhyah Kunthi Wardhani, Andrew E. Grulich, Nurhayati H. Kawi, Yogi Prasetia, Hendry Luis, Gede Benny S. Wirawan, Putu Erma Pradnyani, John Kaldor, Matthew Law, Sudarto Ronoatmodjo, Erik Parulian Sihotang, Pande Putu Januraga, Benjamin R. Bavinton

**Affiliations:** ^1^ Center for Public Health Innovation (CPHI) Udayana University Denpasar Indonesia; ^2^ The Kirby Institute University of New South Wales Sydney New South Wales Australia; ^3^ Klinik Utama Globalindo Jakarta Selatan Indonesia; ^4^ Epidemiology Department, Faculty of Public Health University of Indonesia Depok Indonesia; ^5^ Klinik Bali Medika Badung Indonesia; ^6^ Yayasan Bali Peduli Denpasar Indonesia; ^7^ University of New South Wales Sydney New South Wales Australia; ^8^ Politeknik Kesehatan Kartini Denpasar Indonesia; ^9^ WM Medika Clinic Yayasan Kerti Praja Denpasar Indonesia

**Keywords:** HIV, incidence, Indonesia, MSM, prevalence, TGW

## Abstract

**Introduction:**

There are no longitudinal HIV incidence data among men who have sex with men (MSM) and transgender women (TGW) in Indonesia. We aimed to estimate HIV prevalence and incidence and identify associated factors among clinic attendees in Jakarta and Bali.

**Methods:**

We conducted a retrospective cohort study using medical records from five clinics. We reviewed HIV tests among MSM/TGW aged ≥18 years who attended the clinics between 1 January 2018 to 31 December 2020 in Jakarta and 1 January 2017 to 31 December 2019 in Bali. HIV prevalence was measured at the first test. Those with an HIV‐negative test and ≥1 follow‐up test/s were included in the person‐years (PY) at risk to determine HIV incidence. The PY at risk calculation started at the first negative test until the last recorded negative test or seroconversion. Multivariate Poisson regression was used to determine factors associated with HIV acquisition.

**Results:**

Among 5203 and 2815 individuals with an HIV test result in Jakarta and Bali, respectively, at the first HIV test, 1205 and 616 were HIV positive (HIV prevalence 23.2% and 21.9%). The longitudinal sample included 1418 and 873 individuals, respectively. The median number of tests among repeat testers was 3 in Jakarta (interquartile range [IQR] = 2–4) and 3 in Bali (IQR = 2–5). At baseline, about one‐quarter were aged <25 years, >90% were MSM and >35% had been tested for HIV previously. In Jakarta, there were 127 HIV seroconversions in 1353 PY (incidence 9.39/100 PY, 95% CI = 7.89–11.17), and in Bali, 71 seroconversions in 982 PY (incidence 7.24/100 PY, 95% CI = 5.73–9.13). Compared to those aged 18–24 years, the incidence rate was lower in older patients (Jakarta—30–39 years: aRR = 0.56, 95% CI = 0.34–0.92; 40+ years: aRR = 0.34, 95% CI = 0.14–0.81; Bali—25–29 years: aRR = 0.44, 95% CI = 0.25–0.79; 30–39 years: aRR = 0.33, 95% CI = 0.18–0.61; 40+ years: aRR = 0.06, 95% CI = 0.01–0.48). In Jakarta, incidence was lower in those with university education than in those without (aRR = 0.66, 95% CI = 0.45–0.96). In Bali, those who had been referred by outreach workers had a higher incidence than those who self‐presented for testing (aRR = 1.85, 95% CI = 1.12–3.07).

**Conclusions:**

We observed very high HIV prevalence and incidence rate estimates. Measures to encourage regular testing and effective use of HIV prevention, including pre‐exposure prophylaxis scale‐up and demand creation, are needed.

## 1 INTRODUCTION

In Indonesia, the national prevalence of HIV in the general population remains relatively low at around 0.3% [1]. However, HIV prevalence is much higher in key populations such as men who have sex with men (MSM) and transgender women (TGW), with prevalence estimated in 2019 to be 17.9% and 11.9%, respectively [2]. Although HIV treatments are available at no cost, it was estimated in 2021 that only 28% of people living with HIV accessed them [1], and a 2018 longitudinal study found poor rates of retention in treatment and viral suppression among MSM and TGW [3]. HIV pre‐exposure prophylaxis (PrEP) became available in a limited number of locations across Indonesia in 2022 [4]; however, uptake and retention have been poor [1]. As in other countries, MSM and TGW in Indonesia face stigma and discrimination, which can lead to reduced access to HIV prevention and treatment services [5]. In response, private or non‐government “community‐friendly” clinics for MSM and TGW (and other key populations) have been established, providing a safer space for patients to be tested and treated for HIV [6, 7, 8].

Little is known about HIV incidence rates in Indonesia. To our best knowledge, no retrospective or prospective longitudinal observational cohort study with an HIV diagnosis endpoint has ever been conducted in the MSM and TGW population (or any key population) in Indonesia. Retrospective approaches using pre‐existing medical record (MR) data are commonly used to estimate HIV incidence rates [9]. Advantages of this approach include the ability to estimate HIV incidence rates in clinical settings and the flexibility to identify changes in the incidence trends over time. Furthermore, retrospective methods are relatively inexpensive, making them particularly suitable for low‐resource settings [9]. In this retrospective cohort study, we aimed to estimate the HIV prevalence and incidence rates, and to determine factors associated with these, among patients receiving HIV testing at “community friendly” clinics in Jakarta and Bali.

## 2 METHODS

### 2.1 Study setting and the national HIV testing surveillance documents

This study included data from one clinic in Jakarta and four clinics in Bali. Jakarta and Bali were among the top six provinces with the highest HIV prevalence in Indonesia [10]. All the clinics, which are run by community‐based organizations (aiming to provide inclusive and community‐friendly sexual health services), were known to either target MSM and TGW or be MSM‐ or TGW‐friendly. These sites included: (1) Klinik Utama Globalindo, Jakarta; (2) Klinik Bali Medika, Badung, Bali; (3) Klinik Yayasan Bali Peduli, Denpasar, Bali; (4) Klinik Anggrek Bali Peduli, Gianyar, Bali; and (5) Klinik WM Medika Yayasan Kerti Praja, Denpasar, Bali.

HIV testing occurred according to the national guidelines and involved blood sampling, an initial rapid HIV test and two further rapid HIV tests if the first was reactive [11, 12]. The guidelines recommend that key populations do HIV tests regularly, at least once every year [12]. The national HIV and sexually transmitted infections (STIs) surveillance system mandates all HIV voluntary counselling and testing (VCT) and provider‐initiated testing and counselling (PITC) to be recorded using standardized VCT and PITC forms at each test. Both forms are part of the national notification system “Sistem Informasi HIV/AIDS or SIHA” (HIV/AIDS Information System) and are available as paper‐based and digital forms. In the study clinics, it was common for the paper‐based form to be completed and attached to each client's physical MR, and for the data on the forms to be entered into SIHA afterwards. Along with the clinic MR number, each individual presenting for HIV testing has a unique HIV testing registration ID, which follows a common convention consisting of a 10‐digit code (registration ID). In the participating clinics, the VCT form was commonly used to document HIV testing among key populations and PITC among pregnant women who were referred to the test. For the purposes of this study, we examined the data from participants’ HIV VCT forms.

### 2.2 Study design and population

We conducted a retrospective observational cohort study involving a review of participants’ pre‐existing MR of HIV tests conducted at the five participating clinics. We reviewed MR from all MSM and TGW attending the clinics and who had an HIV test between 1 January 2018 and 31 December 2020 in Jakarta and 1 January 2017 to 31 December 2019 in Bali.

Periodically, as mandated, four clinics with access to SIHA entered the data from the paper‐based VCT forms into locally installed offline versions of SIHA (version 1.7), which was then uploaded monthly to the national online SIHA 1.7 database. We extracted relevant data from these locally installed databases from these four clinics for the 2017–2020 period. However, although using the same paper‐based VCT form as the other four clinics, one clinic in Denpasar did not have access to offline and online SIHA 1.7; instead, the clinic staff entered some variables from the VCT form into the clinic's own electronic database. To extract the relevant data from this one clinic, the clinic staff provided the list of clients who visited for HIV testing between 2017 and 2019 from the clinic's electronic database. Then, the study staff reviewed the paper MR and laboratory reports for the individuals listed to extract and manually enter the remaining data from the VCT forms into an electronic study database.

We created two cohorts for Jakarta and Bali participants. For both Jakarta and Bali, we identified the client's HIV testing registration ID in each clinic and checked for any multiple registration IDs at either the same or different clinics. Eligible participants were included in the baseline cohort, with the baseline visit defined as the date of the first visit during the study period. Participants with ≥1 follow‐up visits were included in the longitudinal cohort. In Bali, participants who attended multiple clinics could be identified across clinics using the client's registration ID. Identifying clinic switches among thousands of MRs was crucial to avoid duplication. HIV tests in the 6 months following 31 December of the relevant final year (2020 for Jakarta, 2019 for Bali) were reviewed to determine if patients were still HIV negative at the end of the relevant final year. All data were censored on 31 December of the relevant final year or at the time of the participant's last HIV test within the study period. As directly indicated on the VCT form, we included individuals with the following inclusion criteria: (1) biologically male at birth (regardless of current gender identity); (2) identified on the VCT form as MSM or TGW; (3) citizen of Indonesia; (4) aged 18 years or older; (5) residing in Jakarta or Bali at the time of the HIV test; and (6) not previously diagnosed with HIV.

### 2.3 Data collection, outcomes and covariates

The outcome variable was confirmed HIV diagnosis. HIV testing results were entered individually for each rapid test, with the combined HIV diagnosis variable having three levels: positive, negative and indeterminate. Covariates from the VCT form included in this analysis were: Clinic; Date of visit; Marital status to a woman (Never, Married, Widowed, Unknown); Location (Jakarta, Bali); Key population (MSM, TGW); Visit status (Voluntary, Referred; a time‐dependent variable whereby referred participants represented individuals attending the HIV test as suggested by their employer/workspace, outreach worker, peer support group, partner or community‐based organization); Motivation for HIV test (Curious to know result, Symptoms, At risk, Retest to confirm after window period; time‐dependent); Condomless vaginal intercourse (CLVI) and the approximate date of the last occasion (Yes, No; time‐dependent); Condomless anal intercourse (CLAI) and the approximate date of the last occasion (Yes, No; time‐dependent); and Needle sharing prior to visit and the approximate date of the last occasion (Yes, No; time‐dependent). Additionally, the following derived covariates were created from the original variables on the VCT form: Visit type (Baseline, Follow‐up); Age group (continuous; 18–24, 25–29, 30–39, 40+); Education (Elementary school or less, Junior and/or senior high school, University, Unknown); With university education (Yes, No); and Employment (Not working, Fixed income, Not‐fixed income, Freelance, Student, Unknown). For time‐dependent variables, we used the relevant data for each visit. We employed multiple imputation‐chained equation techniques to address missing data prior to conducting bivariate and multivariate analyses.

### 2.4 Statistical analysis

**Figure 1:**
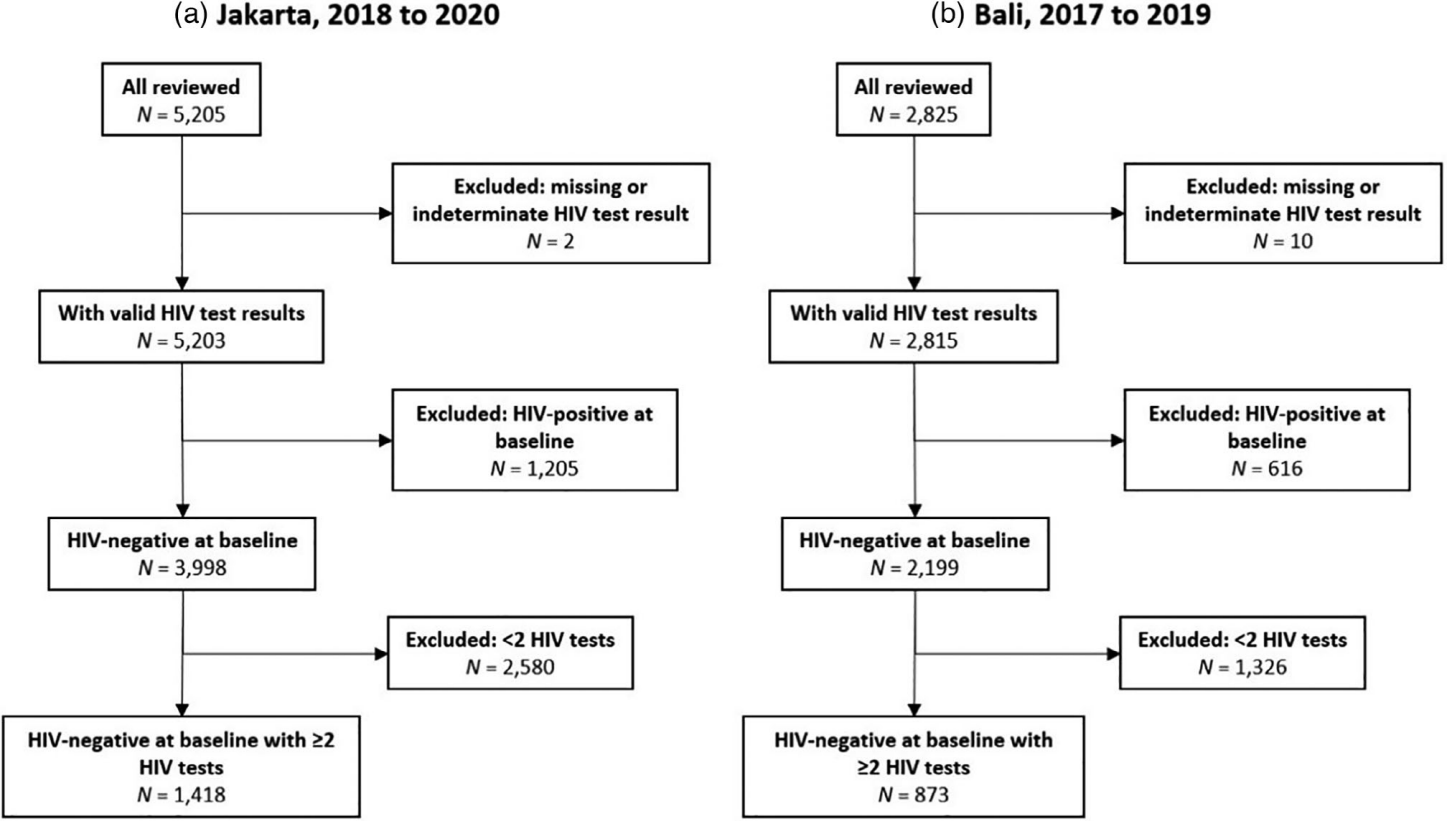
Flowchart of study sample derivation.

Analyses were conducted using Stata version 12.1 (Stata Corporation, College Station, Texas, USA). We determined baseline and longitudinal samples for Jakarta and Bali (see Figure 1). To summarize participants’ characteristics, we calculated the proportion of participants falling into specific categories (e.g. age groups, university education) based on the available data. Participants’ characteristics were calculated for Jakarta and Bali separately, for the HIV‐positive and HIV‐negative participants at baseline, and for those included in the longitudinal sample. Baseline HIV prevalence was estimated for Jakarta and Bali separately, calculated as the number of participants with an HIV‐positive test result divided by the total number of participants. We conducted bivariable and multivariable Poisson regression models to determine factors associated with baseline HIV prevalence using all participants’ baseline data, and we report the prevalence rate ratios (RR), adjusted RR (aRR) and 95% confidence intervals (CI) for these associations.

Using the longitudinal sample, we calculated the HIV incidence rate estimates for Jakarta and Bali separately, which were defined as the number of incident cases divided by the person‐years (PY) of follow‐up. PY at risk was defined as the period between the baseline HIV‐negative test to the last recorded HIV‐negative test or seroconversion, censored on 31 December of the final year. We conducted Poisson regression models to determine factors associated with HIV incidence using participants’ data in the longitudinal cohort. We report incidence rate ratios (RR), adjusted RR (aRR) and 95% CI for these associations. In all the Poisson regression models, covariates with *p*<0.1 in the bivariable models were included in the multivariable model.

### 2.5 Ethical considerations

Human research ethical approval was obtained from: the University of New South Wales, Sydney (HC200874); the Faculty of Medicine, Udayana University/Sanglah Hospital (No.2488/UN14.2.2.VII.14/LT/2020); and the Faculty of Public Health, University of Indonesia (Ket‐17/UN2.F10.D11/PPM.00.02/2022). Individual consent was not obtained from participants. However, organizational approvals were obtained to use and analyse the collected data from each clinic. Participant's records and information were anonymized and de‐identified before analysis.

## 3 RESULTS

The total number of participants whose MRs were reviewed in this study was 5205 and 2825 for Jakarta and Bali, respectively (Figure 1). After excluding participants with missing or indeterminate HIV test results, there were 5203 and 2815 participants for Jakarta and Bali, respectively; these comprised the baseline sample used to determine HIV prevalence. After excluding participants with a positive HIV test result at baseline, there were 3998 and 2199 records with HIV‐negative results at baseline for Jakarta and Bali, respectively. After excluding those without follow‐up visits, 1418 and 873 participants had at least one follow‐up visit in Jakarta and Bali, respectively. Clinic switching in Bali among the participants accounted for 80 out of 873 individuals with valid HIV results (9.2%).

The baseline HIV prevalence was 23.2% (95% CI = 21.9–24.3%) in Jakarta and 21.9% (95% CI = 20.3–23.4%) in Bali. There were few statistically significant differences between those with prevalent HIV at baseline, those who tested HIV negative and those who were included in the longitudinal samples (Table 1). Most participants were aged less than 30 years, were MSM (>90%), had never been married (>90% in Jakarta and >60% in Bali) and were working at the time of the HIV test (about 85%). At baseline, HIV‐negative participants had higher levels of university education (51.8% in Jakarta; 31.2% in Bali) than HIV‐positive participants (41.5% in Jakarta; 27.6% in Bali). The proportion of participants with a history of prior HIV testing was higher among the HIV‐negative participants at baseline (54.1% in Jakarta; 50.0% in Bali) compared to the HIV‐positive participants (43.2% in Jakarta; 35.1% in Bali). In Jakarta, the most common reason for presenting for HIV testing was “curious to know status,” and in Bali, it was “having had a risk episode.” Higher levels of CLVI and CLAI were reported in Jakarta than in Bali.

**Table 1:** Baseline characteristics

	Jakarta (2018–2020)			Bali (2017–2019)		
	Baseline sample: HIV positive (*n* = 1205)	Baseline sample: HIV negative (*n* = 3998)	Cohort sample: HIV negative at baseline (*n* = 1418)	Baseline sample: HIV positive (*n* = 616)	Baseline sample: HIV negative (*n* = 2199)	Cohort sample: HIV negative at baseline (*n* = 873)
Number of tests among repeat testers:	–	–		–	–	
Mean (range)			3.4 (2–9)			4.2 (2–13)
Median (interquartile range [IQR])			3 (2–4)			3 (2–5)
Age group						
18–24 years	336 (27.9)	1093 (27.3)	392 (27.6)	165 (26.8)	594 (27.0)	235 (26.9)
25–29 years	476 (39.5)	1466 (36.7)	532 (37.5)	199 (32.3)	690 (31.4)	295 (33.8)
30–39 years	318 (26.4)	1112 (27.8)	380 (26.8)	187 (30.4)	704 (32.0)	275 (31.5)
40+ years	75 (6.2)	327 (8.2)	114 (8.0)	65 (10.6)	211 (9.6)	68 (7.8)
Key population						
MSM	1186 (98.4)	3762 (94.1)	1312 (92.5)	601 (97.6)	2114 (96.1)	823 (94.3)
TGW	19 (1.6)	236 (5.9)	106 (7.5)	15 (2.4)	85 (3.9)	50 (5.7)
Marital status						
Never	1134 (94.1)	3786 (94.7)	1344 (94.8)	413 (67.1)	1555 (70.7)	671 (76.9)
Currently married	56 (4.7)	156 (3.9)	48 (3.4)	31 (5.0)	79 (3.6)	32 (3.7)
Divorced/widowed	11 (0.9)	48 (1.2)	23 (1.6)	8 (1.3)	16 (0.7)	7 (0.8)
Unknown	4 (0.3)	8 (0.2)	3 (0.2)	164 (26.6)	549 (25.0)	163 (18.7)
Education						
Elementary school or less	15 (1.2)	53 (1.3)	16 (1.1)	11 (1.8)	29 (1.3)	12 (1.4)
Completed junior and/or high school	681 (56.5)	1842 (46.1)	586 (41.3)	309 (50.2)	1029 (46.8)	416 (47.7)
University	500 (41.5)	2071 (51.8)	806 (56.8)	170 (27.6)	685 (31.2)	293 (33.6)
Unknown	9 (0.8)	32 (0.8)	10 (0.7)	126 (20.5)	456 (20.7)	152 (17.4)
Employment						
Not working	25 (2.1)	124 (3.1)	30 (2.1)	43 (7.0)	180 (8.2)	58 (6.6)
Working	1064 (88.3)	3407 (85.2)	1189 (83.9)	446 (72.4)	1520 (69.1)	613 (70.2)
Student	96 (7.8)	397 (9.9)	166 (11.7)	5 (0.8)	20 (0.9)	6 (0.7)
Unknown	20 (1.7)	70 (1.8)	33 (2.3)	122 (19.8)	479 (21.8)	196 (22.5)
Ever had prior HIV test						
No	684 (56.8)	1837 (46.0)	489 (34.5)	400 (64.9)	1100 (50.0)	331 (37.9)
Yes	521 (43.2)	2161 (54.1)	929 (65.5)	216 (35.1)	1099 (50.0)	542 (62.1)
Visit status						
Voluntary	572 (47.5)	2166 (54.2)	854 (60.2)	281 (45.6)	1059 (48.2)	459 (52.6)
Referred	633 (52.5)	1831 (45.8)	563 (39.7)	292 (47.4)	983 (44.7)	357 (40.9)
Unknown	0 (0.0)	1 (0.03)	1 (0.1)	43 (7.0)	157 (7.1)	57 (6.5)
Motivation to test (participants can report >1 motivations)						
Curious to know status	702 (58.3)	2500 (62.5)	796 (56.1)	250 (40.6)	930 (42.3)	441 (50.5)
Symptomatic	53 (4.4)	89 (2.2)	34 (2.4)	18 (2.9)	33 (1.5)	22 (2.5)
Had a risk episode	330 (27.4)	1037 (25.9)	414 (29.2)	445 (72.2)	1438 (65.4)	515 (59.0)
Retest to confirm status	10 (0.8)	24 (0.6)	12 (0.9)	46 (7.5)	395 (18.0)	187 (21.4)
Risk prior to test (participants can report >1 risks)						
Condomless vaginal intercourse (CLVI)	227 (18.8)	637 (15.9)	183 (12.9)	67 (10.9)	203 (9.2)	94 (10.8)
Condomless anal intercourse (CLAI)	1133 (94.0)	3548 (88.7)	1280 (90.3)	431 (70.0)	1518 (69.0)	570 (65.3)
Shared needles	3 (0.3)	4 (0.1)	1 (0.1)	1 (0.2)	2 (0.1)	1 (0.1)

Table 2 presents factors associated with HIV diagnosis at baseline in Jakarta and Bali separately. Focusing on the multivariable analyses, in Jakarta, HIV‐positive test results were lower among those who had university education (aRR = 0.76, 95% CI = 0.67–0.86), had ever had a prior HIV test (aRR = 0.73, 95% CI = 0.65–0.82) and were curious to know their HIV status as their motivation for testing (aRR = 0.86, 95% CI = 0.76–0.96). An HIV‐positive test result was higher among those who were referred to HIV testing (aRR = 1.16, 95% CI = 1.03–1.30), reported experiencing symptoms as the testing motivation (aRR = 1.47, 95% CI = 1.10–1.95), reported CLVI (aRR = 1.17, 95% CI = 1.01–1.35) and reported CLAI (aRR = 1.78, 95% CI = 1.40–2.26). In Bali, an HIV‐positive test result at baseline was lower among those who had ever had a prior HIV test (aRR = 0.70, 95% CI = 0.58–0.84) and reported confirmatory retesting as their motivation to test (aRR = 0.55, 95% CI = 0.39–0.75) and was higher among those who reported HIV risk as the testing motivation (aRR = 1.36, 95% CI = 1.14–1.62).

**Table 2:** Factors associated with baseline HIV prevalence

	Jakarta (2018–2020), *n* = 5203			Bali (2017–2019), *n* = 2815		
	*n*/*N* (prevalence %)	RR (95% CI)	aRR (95% CI)^a^	*n*/*N* (prevalence %)	RR (95% CI)	aRR (95% CI)^a^
Total	1205/5203 (23.2)	–	–	616/2815 (21.9)	–	–
Age group						
18–24 years	336/1429 (23.5)	Ref.	Ref.	165/759 (21.7)	Ref.	–
25–29 years	476/1942 (24.5)	1.04 (0.91–1.20); *p* = 0.560	1.19 (1.03–1.38); *p*‐value = 0.017	199/889 (22.4)	1.03 (0.84–1.27); *p* = 0.781	–
30–39 years	318/1430 (22.2)	0.95 (0.81–1.10); *p* = 0.476	1.05 (0.90–1.23); *p*‐value = 0.540	187/891 (21.0)	0.97 (0.78–1.19); *p* = 0.742	–
40+ years	75/402 (18.7)	0.79 (0.62–1.02); *p* = 0.070	0.85 (0.66–1.10); *p*‐value = 0.220	65/276 (23.6)	1.08 (0.81–1.44); *p* = 0.585	–
Marital status						
Never	1134/4920 (23.1)	Ref.	–	413/1968 (21.0)	Ref.	–
Currently married	56/212 (26.4)	1.14 (0.87–1.49); *p* = 0.348	–	31/110 (28.2)	1.26 (0.89–1.77); *p* = 0.190	–
Divorced/widowed	11/59 (18.6)	0.80 (0.44–1.44); *p* = 0.454	–	8/24 (33.3)	1.38 (0.70–2.70); *p* = 0.352	–
Unknown	4/12 (33.3)	–	–	164/713 (23.0)	–	–
University education						
No	705/2632 (26.8)	Ref.	Ref.	446/1960 (22.8)	Ref.	
Yes	500/2571 (19.5)	0.73 (0.65–0.81); *p*<0.001	0.76 (0.67–0.86); *p*<0.001	170/855 (19.9)	0.87 (0.73–1.04); *p* = 0.134	–
Employment						
Not working	25/149 (16.8)	Ref.	–	43/223 (19.3)	Ref.	–
Working	1064/4471 (23.8)	1.39 (0.94–2.06); *p* = 0.101	–	446/1966 (22.7)	1.16 (0.86–1.57); *p* = 0.341	–
Student	96/493 (19.5)	1.15 (0.74–1.78); *p* = 0.528	–	5/25 (20.0)	1.11 (0.59–2.08); *p* = 0.750	–
Unknown	20/90 (22.2)	–	–	122/601 (20.3)	–	–
Ever had prior HIV test						
No	684/2.521 (27.1)	Ref.	Ref.	400/1500 (26.7)	Ref.	Ref.
Yes	521/2682 (19.4)	0.72 (0.64–0.80); *p*<0.001	0.73 (0.65–0.82); *p*<0.001	216/1315 (16.4)	0.62 (0.52–0.73); *p*<0.001	0.70 (0.58–0.84); *p*<0.001
Visit status						
Voluntary	572/2738 (20.9)	Ref.	Ref.	281/1340 (21.0)	Ref.	–
Referred	633/2464 (25.7)	1.23 (1.10–1.38); *p* = 0.000	1.16 (1.03–1.30); *p* = 0.018	292/1275 (22.9)	1.08 (0.92–1.27); *p* = 0.339	–
Unknown	0/1 (0.0)	–	–	43/300 (21.5)	–	–
Motivation to test: Curious to know status						
No	503/2001 (25.1)	Ref.	Ref.	366/1635 (22.4)	Ref.	–
Yes	702/3202 (21.9)	0.87 (0.78–0.98); *p* = 0.019	0.86 (0.76–0.96); *p* = 0.010	250/1180 (21.2)	0.95 (0.81–1.11); *p* = 0.502	–
Motivation to test: Symptomatic						
No	1152/5061 (22.8)	Ref.	Ref.	598/2764 (21.6)	Ref.	Ref.
Yes	53/142 (37.3)	1.64 (1.25–2.16); *p*<0.001	1.47 (1.10–1.95); *p* = 0.008	18/51 (35.3)	1.63 (1.02–2.61); *p* = 0.041	1.59 (0.99–2.54); *p* = 0.054
Motivation to test: Had a risk episode						
No	875/3836 (22.8)	Ref.	–	171/932 (18.4)	Ref.	Ref.
Yes	330/1367 (24.1)	1.06 (0.93–1.20); *p* = 0.380	–	445/1883 (23.7)	1.29 (1.08–1.54); *p* = 0.005	1.36 (1.14–1.62); *p* = 0.001
Motivation to test: Retest to confirm status						
No	1195/5169 (23.1)	Ref.	–	570/2374 (24.0)	Ref.	Ref.
Yes	10/34 (29.4)	1.27 (0.68–2.37); *p* = 0.448	–	46/441 (10.4)	0.43 (0.32–0.59); *p*<0.001	0.55 (0.39–0.75); *p*<0.001
Risk prior to test: CLVI						
No	978/4339 (22.5)	Ref.	Ref.	549/2545 (21.6)	Ref.	–
Yes	227/864 (26.3)	1.17 (1.01–1.35); *p* = 0.037	1.17 (1.01–1.35); *p* = 0.036	67/270 (24.8)	1.15 (0.89–1.48); *p* = 0.279	–
Risk prior to test: CLAI						
No	72/522 (13.8)	Ref.	Ref.	185/866 (21.4)	Ref.	–
Yes	1133/4681 (24.2)	1.75 (1.38–2.23); *p*<0.001	1.78 (1.40–2.26); *p*<0.001	431/1949 (22.1)	1.04 (0.87–1.23); *p* = 0.694	–

Abbreviations: CLAI, condomless anal intercourse; CLVI, condomless vaginal intercourse.

^a^ Covariates with *p*<0.1 in bivariable models were included in the multivariable models.

In the longitudinal sample of 1318 and 873 participants in Jakarta and Bali, respectively, the median number of HIV tests was 3 in Jakarta (interquartile range [IQR] = 2–4) and 3 in Bali (IQR = 2–5; Table 1). Table 3 shows the HIV incidence rates over 1353 PY of follow‐up in Jakarta and 982 PY in Bali. The HIV incidence rate was 9.39/100 PY (95% CI = 7.89–11.17) in Jakarta and 7.24/100 PY (95% CI = 5.73–9.13) in Bali. In Jakarta, the incidence was 18.27/100 PY in 2018, decreased to 9.16/100 PY in 2019 and remained the same in 2020 (9.0/100 PY). In Bali, the incidence was 11.20/100 PY in 2017, decreased to 7.08/100 PY in 2018 and decreased to 6.40/100 PY in 2019.

**Table 3:** HIV incidence per 100 PY over time, by location

	Jakarta (*n* = 1418)				Bali (*n* = 873)			
	HIV acquisitions/PY	Incidence/100 PY	Lower 95% CI	Upper 95% CI	HIV acquisitions/PY	Incidence/100 PY	Lower 95% CI	Upper 95% CI
Total	127/1353	9.39	7.89	11.17	71/982	7.24	5.73	9.13
2017	–	–	–	–	14/125	11.20	6.63	18.91
2018	9/50	18.27	9.51	35.11	23/325	7.08	4.71	10.66
2019	42/459	9.16	6.77	12.39	34/532	6.40	4.57	8.95
2020	76/845	9.00	7.19	11.27	–	–	–	–

Table 4 shows factors associated with HIV incidence for Jakarta and Bali separately. Focusing on the multivariable analyses, in Jakarta, the HIV incidence rate was lower among those aged 30–39 years (aRR = 0.56, 95% CI = 0.34–0.92) and aged 40 years or older (aRR = 0.34, 95% CI = 0.14–0.81) and who had university education (aRR = 0.66, 95% CI = 0.45–0.96). In Bali, the HIV incidence rate was lower among those aged over 24 years (for those aged 25–29 years: aRR = 0.44, 95% CI = 0.25–0.79; for those aged 30–39 years: aRR = 0.33, 95% CI = 0.18–0.61; for those aged 40+ years: aRR = 0.06, 95% CI = 0.01–0.48), and was higher among those who were referred for HIV testing rather than presenting for testing voluntarily (aRR = 1.85, 95% CI = 1.12–3.07).

**Table 4:** Factors associated with HIV incidence

	Jakarta (2018–2020), *n* = 1418			Bali (2017–2019), *n* = 873		
	*n*/PY (incidence/100 PY)	RR (95% CI)	aRR (95% CI)^a^	*n*/PY (incidence/100 PY)	RR (95% CI)	aRR (95% CI)^a^
Age group						
18–24 years	37/258 (14.35)	Ref.	Ref.	23/165 (13.96)	Ref.	Ref.
25–29 years	52/536 (9.70)	0.66 (0.43–1.00); *p* = 0.049	0.80 (0.51–1.25); *p* = 0.323	26/334 (7.79)	0.44 (0.25–0.79); *p* = 0.006	0.44 (0.25–0.79); *p* = 0.005
30–39 years	32/429 (7.46)	0.47 (0.29–0.75); *p* = 0.002	0.56 (0.34–0.92); *p* = 0.022	20/376 (5.32)	0.33 (0.18–0.60); *p*<0.001	0.33 (0.18–0.61); *p*<0.001
40+ years	6/131 (4.61)	0.31 (0.13–0.72); *p* = 0.007	0.34 (0.14–0.81); *p* = 0.015	2/107 (1.87)	0.07 (0.01–0.50); *p* = 0.008	0.06 (0.01–0.48); *p* = 0.008
Marital status						
Never	120/1274 (9.42)	Ref.	–	55/794 (6.93)	Ref.	–
Currently married	4/54 (7.43)	0.83 (0.31–2.25); *p* = 0.712	–	2/31 (6.56)	0.87 (0.23–3.33); *p* = 0.834	–
Divorced/widowed	3/23 (13.26)	1.28 (0.41–4.04); *p* = 0.670	–	0/7 (0.0)	0.00 (0.00–0.00); *p* = 0.999	–
Unknown	0/3 (0.00)	–	–	14/151 (9.29)	–	–
University education						
No	70/584 (12.01)	Ref.	Ref.	45/622 (7.25)	Ref.	–
Yes	57/770 (7.41)	0.60 (0.42–0.84); *p* = 0.004	0.66 (0.45–0.96); *p* = 0.029	26/361 (7.22)	1.02 (0.62–1.68); *p* = 0.946	–
Employment						
Not working	2/24 (8.38)	Ref.	–	4/33 (12.44)	Ref.	–
Working	101/1152 (8.77)	1.20 (0.30–4.87); *p* = 0.799	–	43/580 (7.43)	0.61 (0.20–1.88); *p* = 0.385	–
Student	20/146 (13.73)	1.89 (0.44–8.11); *p* = 0.391		0/7 (0.0)	0.00 (0.00–0.00); *p* = 1.000	–
Unknown	4/32 (12.88)	–	–	24/364 (6.61)	–	–
Visit status						
Voluntary	92/981 (9.38)	Ref.	–	42/671 (6.26)	Ref.	Ref.
Referred	35/373 (9.41)	1.21 (0.82–1.79); *p* = 0.331	–	27/277 (9.76)	1.78 (1.09–2.90); *p* = 0.021	1.85 (1.12–3.07); *p* = 0.017
Unknown	–	–	–	2/34 (5.94)	–	–
Motivation to test: Curious to know status						
No	48/487 (9.86)	Ref.	–	39/541 (7.22)	Ref.	–
Yes	79/866 (9.13)	0.88 (0.62–1.27); *p* = 0.503	–	32/441 (7.26)	0.88 (0.55–1.43); *p* = 0.616	–
Motivation to test: Symptomatic						
No	122/1319 (9.25)	Ref.	–	70/976 (7.18)	Ref.	–
Yes	5/34 (14.75)	1.61 (0.66–3.94); *p* = 0.299	–	1/6 (17.71)	3.31 (0.46–23.86); *p* = 0.235	–
Motivation to test: Had a risk episode						
No	86/942 (9.14)	Ref.	–	33/504 (6.55)	Ref.	–
Yes	41/412 (9.97)	1.07 (0.74–1.56); *p* = 0.703	–	38/478 (7.96)	1.53 (0.95–2.48); *p* = 0.081	1.11 (0.55–2.21); *p* = 0.771
Motivation to test: Retest to confirm status						
No	124/1320 (9.40)	Ref.	–	42/543 (7.74)	Ref.	–
Yes	3/34 (8.98)	0.94 (0.30–2.97); *p* = 0.921	–	29/439 (6.61)	0.83 (0.51–1.37); *p* = 0.470	–
Risk prior to test: CLVI						
No	113/1208 (9.36)	Ref.	–	67/943 (7.11)	Ref.	–
Yes	14/146 (9.65)	1.13 (0.65–1.97); *p* = 0.670	–	4/39 (10.27)	2.64 (0.96–7.25); *p* = 0.060	2.28 (0.80–6.52); *p* = 0.123
Risk prior to test: CLAI						
No	4/107 (3.76)	Ref.	Ref.	28/455 (6.16)	Ref.	–
Yes	123/1247 (9.87)	2.50 (0.92–6.77); *p* = 0.071	2.45 (0.91–6.64); *p* = 0.078	43/527 (8.16)	1.53 (0.94–2.49); *p* = 0.086	1.56 (0.78–3.14); *p* = 0.211

Abbreviations: CLAI, condomless anal intercourse; CLVI, condomless vaginal intercourse.

^a^ Covariates with *p*<0.1 in bivariable models were included in the multivariable models.

## 4 DISCUSSION

Our retrospective cohort study demonstrated high HIV prevalence and incidence estimates among our participants. We found that, at baseline, HIV‐positive cases were less likely among individuals who had prior HIV testing. Furthermore, at baseline, being referred to a test was found to be associated with being HIV positive. Unsurprisingly, our study also revealed that being curious about one's HIV status was associated with a lower HIV prevalence at baseline. It is important to note that not all clients came to the clinics due to symptoms, as, in our study setting, many clients came for regular tests as required by the Global Fund programme, in which the clinics’ outreach workers were actively engaged with the community to bring them to the clinic for tests regardless of pre‐existing symptoms.

Over time, the HIV incidence estimates in this study evolved, marked by initially high incidence rates in the first year, followed by stabilization or decline in the following 2 years of observation. Bali seemed to have had a more consistent decrease in HIV incidence, with a larger decline from 2017 to 2019 than Jakarta. Jakarta had a greater decline in the estimated HIV incidence from 2018 to 2019 than Bali. However, it is worth noting that Jakarta had a higher HIV incidence rate in 2018 compared to Bali, so the relative decline in the estimates might be different when compared to a similar baseline period. Bali had a lower incidence rate in the preceding years; however, to infer that Bali has a better HIV control programme or a relatively low burden than Jakarta, more data and information about the context and interventions in both locations are required to draw a definitive conclusion. In addition, it is important to note that this is just a 1‐year comparison (2018–2019), and a longer time frame is needed to evaluate the effectiveness of HIV control programmes in both locations. It is also important to note that one of the potential contributors to the decrease was the improvement in the testing and treatment coverage. HIV incidence in 2019 and 2020 were similar in Jakarta at 9.16/100 PY and 9.0/100 PY, respectively. It was unclear how the COVID‐19 pandemic affected the 2020 HIV incidence rate at this clinic. However, some Indonesian studies have identified how the pandemic significantly impacted access to HIV‐related services among MSM and TGW in Indonesia. Fear of contracting COVID‐19, lack of information about service provision during the pandemic, financial difficulties and long‐distance travel to clinics acted as barriers to accessing HIV services for these vulnerable populations [13, 14, 15, 16].

During the study period, HIV incidence was 7–9 per 100 PY in Jakarta and Bali, with higher incidence rates observed among younger participants, those without university education. Aligned with two Thailand studies, which indicated that a longer duration of schooling was strongly protective against HIV acquisition through the increased awareness of HIV testing [17, 18], our study has also indicated that higher education is associated with lower HIV acquisition.

We also identified 9.2% of individuals with clinic switches in Bali. Further research should be conducted to better understand the reasons for clinic switching. However, some studies have indicated that convenience, including being community‐friendly, proximity to the venue, stigma, discrimination, confidentiality and healthcare access, likely influenced clinic choice [19, 20, 21, 22]. Although we did not capture data on clinic switching in Jakarta, we could expect that similar dynamics may apply there. The fact that the Jakarta clinic requires some out‐of‐pocket costs for clients and has long clinic hours (9 AM–9 PM, longer than the state‐owned primary healthcare hours where services are provided free‐of‐charge), could impact clinic choice, either switching to our Jakarta clinic or from it.

To the best of our knowledge, our study is the first longitudinal study to follow an HIV‐negative cohort of MSM and TGW using standardized routinely collected data, estimating both baseline prevalence and HIV incidence, in Indonesia. Prior to this study, there was one longitudinal prospective study on HIV‐positive key population cohorts in Indonesia [3]. In Indonesia, most HIV studies have been cross‐sectional [2, 23, 24, 25, 26, 27]. We found a few regional cohort studies from the Philippines and Thailand, which have shown similar findings regarding HIV incidence and prevalence among MSM and TGW. From a 2022 study in Metro Manila, it was stated that there was an HIV incidence of 2.7/100 PY among carefully selected MSM participants [28]. One Thailand study in 2015 showed a rate of 5.5/100 PY among its MSM participants [29]. Meanwhile, prior to Thailand's national PrEP programme, one Thailand study showed an overall HIV incidence rate of 4.8/100 PY with a peak of 6.4/100 PY in the second quarter of 2011 [30]. Furthermore, a 2020 study from an MSM community clinic in Bangkok found an overall incidence rate of 4.1/100 PY, with a more concerning rate among its 13‐ to 22‐year‐old population at 10.0/100 PY [31].

The baseline analysis of HIV prevalence in this study revealed that the participating clinics primarily attracted young, single, employed MSM below the age of 40, most of whom were aged 25–29 and had engaged in CLAI. In Jakarta, we have indicated that HIV diagnoses at baseline were more common among those who reported engaging in condomless sex. A 2018 study in Bali demonstrated relatively high PrEP acceptance among MSM and TGW participants [32], and it is fair to suggest that it is crucial to provide this population with PrEP [33, 34, 35].

Our study results should be considered in light of some limitations. First, this analysis utilized routinely collected data from real‐world clinical settings where patients were not actively followed up, leading to a high proportion of patients with only one (baseline) HIV test visit included in the analysis. The impact of loss‐to‐follow‐up on the estimated HIV incidence is uncertain and may result in an overestimate due to sampling from repeat testers who may be at higher risk. However, it could also be underestimated because the study participants who had prior HIV testing were less likely to acquire HIV. Additionally, the sample consisted of individuals with access to HIV testing, who might be more health‐conscious and at lower risk of acquiring HIV compared to those not included in the sample. Second, clinic‐based cohorts can exhibit selection bias because people seeking out testing may be more likely to be symptomatic for HIV or other STIs. Furthermore, the cohorts were likely to overrepresent individuals engaging in higher levels of risk behaviours (e.g. more sexually active). Third, study data were obtained from a standardized VCT form regularly used in clinic practice, making data merging from all clinics relatively straightforward. However, there were often incomplete variables in the paper‐based documents compared to the electronic data, despite using the same form for both. As a result, several key variables had missing data, and some variables (e.g. socio‐demographic data, detailed sexual behaviours with different partner types, recreational drug use, STIs and PrEP or post‐exposure prophylaxis use) were not investigated. Fourth, self‐reported variables may have been subject to recall and social desirability biases. Fifth, the data were from 2017 to 2020, which may not capture current trends in HIV incidence in Indonesia. Finally, this study only included MSM/TGW participants from a small number of clinics located mainly in urban areas of Jakarta and Bali, which may not represent the overall MSM/TGW population in Indonesia. In particular, as MSM comprised over 95% of the study participants, it may not be possible to generalize the findings to TGW. Therefore, we also suggest further research on this topic, specifically among TGW in Indonesia.

## 5 CONCLUSIONS

To our knowledge, we have provided the first HIV incidence estimates for MSM and TGW in Indonesia arising from a longitudinal cohort study and found them to be concerningly high. Additionally, our findings showed higher HIV prevalence in these populations than found in the 2019 national estimates [2]. The identified demographic and other factors associated with the high prevalence and incidence underscore the urgent need to strengthen and expand existing HIV prevention programmes in Indonesia. Local and international efforts must prioritize greater investment in HIV prevention and treatment for these populations to achieve global AIDS elimination goals. Effective strategies should include increasing HIV prevention awareness for younger generations by using age‐appropriate methods, promoting regular and early testing, expanding PrEP awareness and demand creation programmes, scaling up PrEP implementation and improving key population outreach support to achieve better outreach outcomes.

## COMPETING INTERESTS

BRB has received research grants and honoraria from ViiV Healthcare and Gilead Sciences. The other authors have declared no conflict of interest. The funders had no role in the study's design, the collection, analysis, or interpretation of data, the writing of the manuscript, or the decision to publish the results.

## AUTHORS’ CONTRIBUTIONS

Conceptualization, PPJ, BRB, AEG; methodology, PPJ, BRB, AEG; formal analysis, BRB, BDKW; writing—original draft preparation, BRB, BDKW; writing—review and editing, BDKW, AEG, NHK, YP, HL, GBSW, PEP, JK, ML, SR, EPS, PPJ, BRB. All authors have read and agreed to the published version of the manuscript.

## FUNDING

This research was co‐funded by a seed grant from the Kirby Institute, University of New South Wales, Sydney, Australia, and the Australian Government through the Australian Alumni Grant Scheme administered by Australia Awards in Indonesia and UNAIDS Indonesia. The corresponding author had full access to the dataset in the study and had final responsibility for publication submission decisions.

## Data Availability

The data that support the findings of this study are available on request from the corresponding author. The data are not publicly available due to privacy or ethical restrictions.
